# Intermolecular Communication in *Bacillus subtilis*: RNA-RNA, RNA-Protein and Small Protein-Protein Interactions

**DOI:** 10.3389/fmolb.2020.00178

**Published:** 2020-08-07

**Authors:** Inam Ul Haq, Peter Müller, Sabine Brantl

**Affiliations:** Matthias-Schleiden-Institut, AG Bakteriengenetik, Friedrich-Schiller-Universität Jena, Jena, Germany

**Keywords:** sRNA, small regulatory RNA, *Bacillus subtilis*, type I toxin-antitoxin system, T-box riboswitch, RNA chaperones, small proteins, antiterminators

## Abstract

In bacterial cells we find a variety of interacting macromolecules, among them RNAs and proteins. Not only small regulatory RNAs (sRNAs), but also small proteins have been increasingly recognized as regulators of bacterial gene expression. An average bacterial genome encodes between 200 and 300 sRNAs, but an unknown number of small proteins. sRNAs can be *cis*- or *trans*-encoded. Whereas *cis*-encoded sRNAs interact only with their single completely complementary mRNA target transcribed from the opposite DNA strand, *trans*-encoded sRNAs are only partially complementary to their numerous mRNA targets, resulting in huge regulatory networks. In addition to sRNAs, uncharged tRNAs can interact with mRNAs in T-box attenuation mechanisms. For a number of sRNA-mRNA interactions, the stability of sRNAs or translatability of mRNAs, RNA chaperones are required. In Gram-negative bacteria, the well-studied abundant RNA-chaperone Hfq fulfils this role, and recently another chaperone, ProQ, has been discovered and analyzed in this respect. By contrast, evidence for RNA chaperones or their role in Gram-positive bacteria is still scarce, but CsrA might be such a candidate. Other RNA-protein interactions involve tmRNA/SmpB, 6S RNA/RNA polymerase, the dual-function aconitase and protein-bound transcriptional terminators and antiterminators. Furthermore, small proteins, often missed in genome annotations and long ignored as potential regulators, can interact with individual regulatory proteins, large protein complexes, RNA or the membrane. Here, we review recent advances on biological role and regulatory principles of the currently known sRNA-mRNA interactions, sRNA-protein interactions and small protein-protein interactions in the Gram-positive model organism *Bacillus subtilis*. We do not discuss RNases, ribosomal proteins, RNA helicases or riboswitches.

## Introduction

The first regulatory RNAs were identified and intensively studied as control elements in replication, conjugation and maintenance of bacterial plasmids as well as in transposition and transduction (rev. in Wagner and Romby, 2015). However, only after the publication of a variety of regulatory RNAs from intergenic regions of *E. coli* by two groups (Argaman et al., 2001; Wassarman et al., 2001), knowledge on bacterial regulatory RNAs started to expand. This was due to systematic bioinformatic approaches combined with experimental studies as well as RNA sequencing, which revealed that an average bacterial genome encodes between 200 and 300 small regulatory RNAs (sRNAs). The majority of sRNA targets are mRNAs. Therefore, sRNA-mRNA interactions play an important role in virtually all bacterial cells. Furthermore, uncharged tRNA can interact with the T-box in 5′ UTRs of various mRNAs encoding amino-acid related genes resulting in transcriptional read-through. Bacterial cells contain a number of RNA chaperones that stabilize sRNAs, modulate mRNA translation or promote mRNA-sRNA interactions. In general, Hfq (rev. in Kavita et al., 2018), ProQ (rev. in Olejniczak and Storz, 2017) and CsrA (rev. in Vakulskas et al., 2015) are known and have been investigated in great detail in this respect. Interestingly, Gram-positive bacteria do not encode ProQ, and Hfq does not seem to play a comparable role as in Gram-negative bacteria. Furthermore, CsrA has not been found to sequester small RNAs in Gram-positives. Other RNA-protein interactions involve tmRNA, the ubiquitous RNase P, 6S RNA that interacts with RNA polymerase, RNA antiterminators and terminators that are bound and regulated by proteins as well as the aconitase, a TCA enzyme that moonlights at iron limiting conditions as RNA binding protein. In addition, interactions between small proteins and larger proteins or protein complexes play an important role in gene expression control. Only a few interactions have been elucidated and investigated in some detail in this new field, e.g., modulation of the function of individual proteins or activation/inactivation of multiprotein complexes by small proteins, interactions of small proteins with membrane proteins or the membrane itself.

Here, we present an overview of recent advances in sRNA/tRNA-mRNA, RNA-protein and small protein-protein interactions in the Gram-positive model organism *Bacillus subtilis*. However, we will not include RNases, RNA helicases, ribosomal proteins or riboswitches.

## RNA-RNA Interactions

Two main groups of interactions will be reviewed here: small regulatory RNAs (sRNAs) that interact with their target mRNAs and tRNAs that interact with the 5′ UTR of mRNAs in the so-called T-box riboswitches.

sRNAs can be classified in *cis*- and *trans*-encoded sRNAs. The first sRNAs discovered 35–40 years ago on plasmids and transposons are *cis*-encoded, i.e., transcribed convergently to their RNA targets from the complementary DNA strand and able to form complete duplexes with them (rev. in Brantl, 2012; Wagner and Romby, 2015). Therefore, each *cis*-encoded sRNA has only one target RNA. By contrast, *trans*-encoded sRNAs are encoded in another region of the genome and are only partially complementary to their broad variety of target RNAs. Whereas for the majority of the currently known ≈150 *E. coli* sRNAs targets have been identified and mechanisms of action elucidated, only a dozen of the 108 confirmed *B. subtilis* sRNAs (Rasmussen et al., 2009; Irnov et al., 2010) have been investigated in great detail. Here, we focus on sRNAs with known targets.

### *Cis*-Encoded sRNAs

The largest group of currently known *B. subtilis cis*-encoded sRNAs are type I antitoxins that interact with their target toxin mRNAs either at their 5′ or 3′ end by a base-pairing mechanism. In addition, the targets for a few other *cis*-encoded sRNAs have been identified and characterized (see below). Table 1 provides an overview of all currently known *cis*-encoded sRNAs in *B. subtilis*.

**TABLE 1:** Overview of *cis*-encoded sRNA/mRNA systems from *B. subtilis.*

**Sense/as gene pair**	**sRNA length**	**sRNA action**	**Target function**	**Biological role/regulation in**	**Regulation peculiarity**
*txpA*/RatA	222 nt	RD	Toxin (59 aa)	Skin maintenance	Glucose dependent, III**
*bsrG*/SR4	180 nt	TI + RD	Toxin (38 aa)	SPβ maintenance?	Temperature dependent
*bsrE*/SR5	163 nt	RD	Toxin (30 aa)	Anaerobic stress	Multistress-responsive
*yonT/yoyJ*/SR6	100/215* nt	RD *yonT* TI *yoyJ*	Toxin (58 aa) Toxin (83 aa)	n. d.	Multistress-responsive, SR6 very stable, III**
*bsrH/as-bsrH*	200 nt	RD	Toxin (29 aa)	n. d.	Multistress-responsive J1 cleaves mRNA
*yabE/*S25	1350 nt^#^	n. d.	Autolysin	Regulation of autolysin expression	sRNA under σ^X^ and σ^M^ control
*gdpP*/S1559	667 nt^#^	TI?	c-di-AMP PD	Regulation of c-diAMP phosphodiesterase	sRNA under σ^D^ control
*cwlO/*S1326	700–2200 nt	n. d.	Endopeptidase type autolysin	Exit from stationary phase	antisense sRNA under σ^B^ control
*rpsD*/S1136^+^	185–600 nt	T interference	Ribosomal protein S4	Ribosome reduction under ethanol stress	sRNA under σ^B^ control dual-function sRNA
*opuB/*S1290.	300–3800 nt	T interference	Choline transporter	Delayed osmoprotection under salt stress	sRNA under σ^B^ control

*RD, promotion of mRNA degradation; PD, phosphodiesterase; TI, translational inhibition; T intereference, transcriptional interference; ?, proposed but not experimentally shown.* *Longer SR6 species due to read-through of SR6 terminator. ^#^Length has not been determined in Northern blots, but was estimated from transcriptome data; III^∗∗^, RNase III is essential for inhibition by the antitoxin; n.d., not determined; ^+^Has been renamed SR7 (Ul Haq et al., 2020) (see bellow). For further details see text.

### Antitoxins in Type I Toxin-Antitoxin Systems

Type I toxin-antitoxin (TA) systems are composed of two elements, a hydrophobic toxin and an RNA antitoxin that neutralizes toxin action by interacting with the toxin mRNA to affect its stability and/or translation. Out of 14 predicted type I TA systems of *B. subtilis* (Durand et al., 2012a), four have been verified experimentally and analyzed in some detail: *txpA*/RatA (Silvaggi et al., 2005), *bsrG*/SR4 (Jahn et al., 2012), *bsrE/*SR5 (Meißner et al., 2015; Müller et al., 2016), and *yonT*/SR6 (Durand et al., 2012b; Reif et al., 2018). The majority of them are located on prophage elements or phage remnants in the chromosome.

**FIGURE 1:**
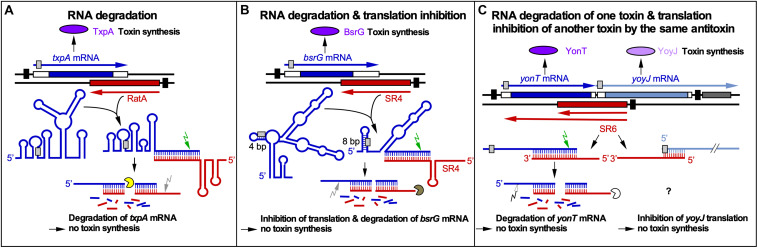
RNA-RNA interactions of *cis*-encoded sRNAs from *B. subtilis*. Black bars denote promoters. Toxins are drawn in purple or light purple, toxin mRNAs in blue or gray blue and antitoxins in red. Toxin ORFs are represented by blue and gray blue bars. Light gray boxes depict RBS. Arrows symbolize endoribonucleases (RNase III, green; RNase Y, gray; unknown RNase, white) and circular segments 3′-5′ exoribonucleases (PNPase, yellow; RNase R, brown; unknown RNase, white). **(A)** Promotion of RNA degradation. The antitoxin RatA and its *txpA* toxin mRNA base-pair at their 3′ ends. **(B)** RNA degradation and translation inhibition. The antitoxin SR4 and the corresponding *bsrG* toxin mRNA interact at their 3′ ends. SR4 binding to *bsrG* mRNA induces a conformational alteration that extends the region sequestering the SD sequence from 4 to 8 bp which inhibits *bsrG* translation. Additionally, the SR4/*bsrG* mRNA interaction facilitates toxin mRNA decay by an initial RNase III cleavage followed by subsequent RNase R and RNase Y degradation. **(C)** One antitoxin inhibits two toxins by different mechanisms. Antitoxin SR6 and *yonT* toxin mRNA base-pair at their 3′ ends, which promotes *yonT* mRNA decay by an initial RNase III cleavage that is followed by degradation by so far unidentified RNases. Furthermore, SR6 interacts with *yoyJ* toxin mRNA by base-pairing at the 5′ ends, which does not promote *yoyJ* mRNA degradation, but prevents *yoyJ* overexpression, possibly by translational inhibition. (Adapted with permission from Brantl and Müller, 2019).

The ***txpA/*RatA** module is encoded on the chromosomal *skin* element. In *ratA* knockout strains, the toxin TxpA (59 aa) causes cell lysis on agar plates after 5 days. The 220 nt long RatA overlaps the 3′ end of *txpA* mRNA by ∼120 nt. The interaction between both RNAs promotes the degradation of *txpA* mRNA by RNase III, which is required for the viability of *B. subtilis* and makes RNase III an essential enzyme in this bacterium (Durand et al., 2012b; Figure 1A). The secondary structures of RatA and *txpA* RNA as well as their complex have been experimentally determined. The *SD* sequence of *txpA* is sequestered in a 5 bp double-stranded region. RatA binding does not alter the *txpA* structure around the *SD* sequence, i.e., it has no influence on its accessibility to the ribosomal 30S SU. None of the interacting loops contains a U-turn motif (see below).

The ***bsrG*/SR4** module is located on the chromosomal SPβ prophage. In *sr4* knockout strains, the hydrophobic toxin BsrG (38 aa) causes cell lysis on agar plates after overnight incubation at 37°C (Jahn et al., 2012, 2015). The 294 nt long *bsrG* mRNA and the 180 nt long antitoxin SR4 interact at their 3′ ends resulting in degradation of *bsrG* mRNA by RNase III 8 nt downstream from the stop codon. RNase III is, however, not involved in the degradation of either *bsrG* RNA or SR4 alone. Endoribonuclease Y and the 3′-5′ exoribonuclease R are responsible for further degradation of both RNAs. PNPase processes SR4 precursors into the mature RNA. In contrast to *txpA*/RatA, RNase III is not essential for the *bsrG*/SR4 system because a Δ*rnc* strain neither lysed on agar plates nor had mutations in the *bsrG* ORF. Furthermore, the RNA chaperone Hfq is not required for the function of the *bsrG*/SR4 system, since a Δ*hfq* strain does neither lyse on agar plates nor displays changed half-lives of SR4 or *bsrG* mRNA (Jahn et al., 2012). The secondary structures of SR4 and *bsrG* mRNA and their complex were experimentally determined (Jahn and Brantl, 2013). SR4 induces structural alterations around the SD sequence of *bsrG* by extending a 4 bp double-stranded region that makes the SD barely accessible into an 8 bp region which inhibits *bsrG* translation. SR4 is, therefore, the first dual-function type I antitoxin: It facilitates *bsrG* mRNA degradation and prevents *bsrG* translation by impairing ribosome access to the *bsrG* SD (Figure 1B).

**FIGURE 2:**
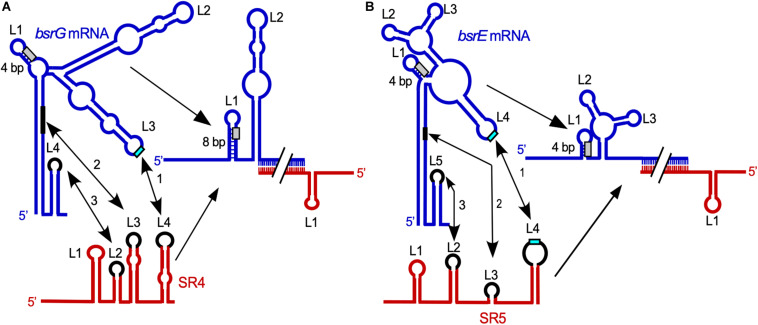
Comparison of the SR4/*bsrG* RNA **(A)** and SR5/*bsrE* RNA **(B)** interaction pathways. Blue, toxin mRNAs; red, RNA antitoxins. U-turn motifs are indicated in turquoise and RBS by light gray boxes. The interaction chronology is designated by 1–3. L, loop. **(A)** The initial contact between SR4 and *bsrG* RNA occurs between L4 of SR4 and L3 of *bsrG* RNA (1). It is followed by helix progression to an interaction between SR4 L3 and the 3′ part of helix P1 of *bsrG* RNA (2) and finally reaches L2 of SR4 that binds terminator loop L4 of *bsrG* RNA (3). The latter interaction is not essential. **(B)** The binding pathway of SR5 and *bsrE* RNA comprises three similar subsequent interactions. Schematic secondary structures are based on experimentally probed structures in Jahn and Brantl (2013) and Meißner et al. (2015). (Adapted with permission from Brantl and Jahn, 2015; Brantl and Müller, 2019).

Complex formation assays with *bsrG* RNA and SR4 yielded an apparent binding constant *k*_*app*_ of 6.5 × 10^5^ M^–1^ s^–1^ (Jahn and Brantl, 2013). The elucidation of the binding pathway of *bsrG* mRNA and SR4 (Jahn and Brantl, 2013; Figure 2A) revealed that binding initiates with a single loop-loop contact between loop L3 of *bsrG* RNA and loop L4 of the SR4 terminator stem-loop. Subsequently, base-pairing progresses via the single-stranded region between L4 and L3 toward L3 of SR4, and, finally to L2 that pairs with the *bsrG* terminator-stem-loop. However, the latter interaction is not essential for efficient binding. A 5′ YUNR motif present in L3 of *bsrG* RNA might form a U-turn (see Heidrich and Brantl, 2003) to provide a scaffold for the efficient initial interaction with SR4. An excess of antitoxin over toxin mRNA is obtained by the 6- to 10-fold higher promoter strength of the *sr4* promoter compared to the *bsrG* promoter (Jahn et al., 2012).

The multistress-responsive ***bsrE/*SR5** module is encoded on the prophage-like element P6. In contrast to *txpA*/RatA and *bsrG*/SR4, deletion of the *sr5* promoter to prevent antitoxin expression does not lead to cell lysis on agar plates. Only *bsrE* overexpression from a multicopy plasmid inhibits cell growth and causes lysis on agar plates indicating that BsrE (30 aa) is a much weaker toxin than BsrG (Müller et al., 2016). SR5 (163 nt) and *bsrE* mRNA (256 nt) interact by 114 complementary bp at their 3′ ends, and this interaction causes degradation of the toxin mRNA by RNase III at all three *bsrE* stop codon positions (Meißner et al., 2015). The secondary structures of *bsrE* mRNA, the antitoxin SR5 and their complex were mapped and are similar to those of *bsrG* RNA and SR4 (Meißner et al., 2015). Complex formation between toxin mRNA and RNA antitoxin were studied and showed a similar k*_*app*_* value of 1–3 × 10^6^ M^–1^ s^–1^. Likewise, the SR5/*bsrE* mRNA interaction pathway (Figure 2B) closely resembles that of *bsrG* RNA and SR4 (see above). The main three differences between both pathways are: (i) the presence of only one U-turn motif in loop L3 of *bsrG* mRNA but two U-turn motifs, one in L4 of *bsrE* RNA and another in terminator loop L4 in SR5 which are engaged in the initial contact, (ii) two stem-loops of SR5, SL2 and SL4, are fundamental for formation of a stable duplex with *bsrE* mRNA, whereas only one stem-loop and a single stranded region of SR4 sufficed and (iii) SR5 binding did not trigger a structural change around the toxin mRNA RBS, whereas SR4 binding did. Therefore, the antitoxin SR5 is monofunctional: It promotes toxin mRNA degradation but does not inhibit *bsrE* translation directly. Interestingly, both *bsrE* mRNA and SR5 respond to different stress factors: Whereas *bsrG* mRNA is only heat-shock sensitive due to its refolding at 48–55°C which results in rapid degradation by RNases Y and J1 (Jahn and Brantl, 2016), *bsrE* mRNA is sensitive to heat-shock, ethanol stress and alkaline pH (Müller et al., 2016). SR5 amounts were influenced by iron limitation, acid, alkaline and anaerobic stress. Oxygen deficiency was the only stress that altered the SR5/*bsrE* mRNA ratio from 9: 1 to 0.5: 1, i.e., has the potential to induce cell lysis (Müller et al., 2016).

The multistress-responsive ***yonT*/*yoyJ*/SR6** module is the second type I TA system encoded on the SPβ prophage. The peculiarity in this system is that antitoxin SR6 interacts with two toxin mRNAs encoding YonT (59 aa) and YoyJ (83 aa). The 3′ end of the 100 nt long SR6 interacts with the 3′ end of *yonT* mRNA to promote its degradation by RNase III. On the other hand, the 5′ end of SR6 binds to the 5′ end *yoyJ* mRNA, but neither affects the amount nor half-life of *yoyJ* RNA. Instead, it seems to inhibit *yoyJ* translation (Reif et al., 2018; Figure 1C). The latter interaction can only be postulated as there is only indirect evidence for YoyJ being a weak toxin: A *yoyJ* overexpression plasmid could only be established by transformation in a *B. subtilis* strain that carries the *sr6* gene in the chromosome (Reif et al., 2018). In contrast to TxpA, BsrG or BsrE, YonT is a very strong toxin, as neither SR6 could be deleted from the chromosome nor *yonT* overexpressed from a medium copy plasmid in *E. coli* or *B. subtilis*. So far, no secondary structures were probed or complex formation studies performed. However, calculations of the *yonT* mRNA and SR6 amounts revealed only in minimal CSE medium with glucose at stationary phase an excess of *yonT* mRNA over SR6, i.e., under these conditions the toxin could be expressed.

Similarly to *bsrE*/SR5, *yonT/yoyJ*/SR6 responds to a number of stress factors: After ethanol stress, *yonT* mRNA disappeared as *bsrE* mRNA within 0.5 min, and heat-shock caused a 4-fold decrease of *yonT* RNA levels. However, in contrast to SR4 or SR5, SR6 levels also decreased about 4-fold after heat-shock. Vancomycin (cell-wall stress) treatment led to a decrease of both *yonT* mRNA and SR6.

Another potential type I TA system is *bsrH/*anti-bsrH (Durand et al., 2012a, b) located on the *skin* prophage. *bsrH* mRNA (285 nt) and anti-bsrH (200 nt) can interact at their 3′ ends and RNase J1 is involved in cleavage of the complex. But so far, it has not been shown that BsrH (29 aa) acts as a toxin in *B. subtilis* and that anti-bsrH neutralizes toxin action.

### Other *Cis*-Encoded sRNAs in *B. subtilis*

At least five other *cis*-encoded sRNAs (antisense RNAs) have been reported: S25, gdpPas/S1559, S1326, S1136-1134 and S1290. **S25** (≈1350 nt) is expressed under control of extracytoplasmic sigma factors *σ*^X^ and *σ*^M^ and regulates an autolysin encoded by *yabE* (Eiumphungporn and Helmann, 2009). **S1559/gdpPas** (Luo and Helmann, 2012) is transcribed under control of s^D^ from the middle of the *gdpP* gene encoding the phosphodiesterase responsible for degradation of cyclic di-AMP. Neither sense nor antisense RNA were detectable in Northern blots. The presence of S1559/gdpPas reduced the level of FLAG-tagged GdpP about 2.5 to three-fold, but could not be associated with any phenotype which makes the biological role of this regulation elusive. Antisense RNA **S1326** is transcribed from a *σ*^B^ dependent promoter located downstream of the *cwlO* gene that encodes a D,L-endopeptidase type autolysin (Noone et al., 2014). S1326 has a heterogenous length (between 700 and 2200 nt) and was detectable under phosphate stress. The largest S1326 transcript extends beyond the *cwlO* promoter. The *cwlO* mRNA is highly unstable due to an RNase Y cleavage in the leader region which seems to be decisive for regulation of this RNA. S1326 influenced weakly the exit of the cells from stationary phase.

The *σ*^B^ dependent sRNA **S1136-1134** transcribed *in cis* to *rpsD* mRNA encoding the ribosomal primary binding protein S4 (22.7 kD) was reported to decrease the amount of *rpsD* mRNA and, consequently, the amount of the small ribosomal subunit ∼1.5 fold under ethanol-stress (Mars et al., 2015a). S1136-1134 could only act *in cis*, which suggests that its mechanism of action is transcriptional interference.

The same holds true for *σ*^B^-dependent **S1290** that is transiently transcribed in response to salt stress. S1290 acts solely *in cis* and independent of RNase III on its convergently transcribed target *opuB* mRNA encoding a choline transporter (Rath et al., 2020). It causes a time-delayed osmotic induction of *opuB* mRNA, since under acute salt stress, *B. subtilis* initially relies on the promiscuous OpuC transporter to import pre-formed compatible solutes as proline- or glycine betaine before employing OpuB to import choline that has to be converted into glycine betaine. In addition to S1290, *opuB* expression is dependent on the degree of the imposed osmotic stress.

For neither of these antisense RNAs, secondary structures or – in case they act through complex formation with their target RNAs – those of the antisenseRNA/target RNA complexes were determined.

### *Trans*-Encoded sRNAs in *B. subtilis*

**FIGURE 3:**
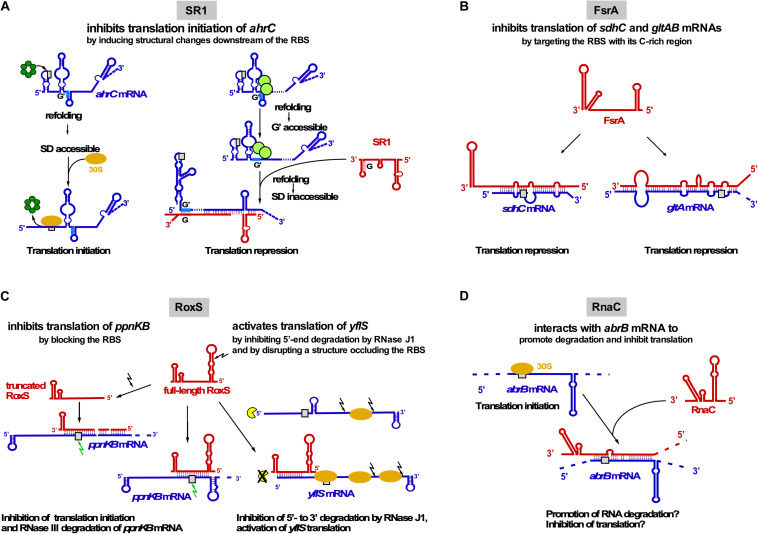
Interactions of *trans*-encoded sRNAs with their target mRNAs **(A)** SR1 interacts with *ahrC* mRNA about 100 nt downstream of the RBS which induces structural changes around the *ahrC* RBS that inhibit translation initiation. Left: Hfq (dark-green) binds immediately upstream of the *ahrC* RBS (gray rectangle) to make it accessible to the 30S SU. Right: By binding to GGA motives 1–3 of *ahrC* mRNA, CsrA (light-green) induces a slight structural change that makes region G′ accessible to complementary region G of SR1. SR1 binding induces a structural change that renders the *ahrC* RBS inaccessible to 30S binding, thereby inhibiting translation initiation (Brantl and Brückner, 2014). **(B)** Interaction of FsrA with *sdhC* and *gltAB* mRNAs. FsrA has numerous targets, but probably for all of them (see text) inhibits translation by targeting the RBS or an adjacent region. **(C)** Interaction of RoxS with two target mRNAs. Whereas RoxS and its truncated derivative repress *ppnKB* translation, full-length RoxS activates *yflS* translation, mainly by inhibiting RNase J1 degradation from the 5′ end. **(D)** Interaction of RNAC with *abrB* mRNA which might promote RNA degradation and inhibit *abrB* translation. Red, sRNAs; blue, target mRNAs; beige oval, 30S SU; gray box, RBS; green arrow, RNase III; black arrow, RNase Y; yellow, RNase J1.

Currently, four *trans*-encoded sRNAs are known for which targets have been identified: SR1 (Licht et al., 2005; Heidrich et al., 2006), FsrA (Gaballa et al., 2008), RoxS (Durand et al., 2015, 2017), and RNAC (Mars et al., 2015b; Figure 3). Among them, SR1 is the only dual-function sRNA which acts as a base-pairing sRNA and as mRNA encoding a small peptide, SR1P (see below). Table 2 provides an overview of all currently known *trans*-encoded sRNAs in *B. subtilis*.

**TABLE 2:** Overview of trans-encoded sRNAs from *B. subtilis.*

**sRNA (nt)**	**Target RNA(s)**	**Biological function**	**Mechanism of action**	**Control of expression/specific characteristic**
SR1 (205)	*ahrC*	Arginine catabolism	TI	CcpN, CcpA, sporulation
FsrA (84)	*sdhCAB*,	Iron sparing response	TI	Fur, iron;
	*citB*	Aconitase		some targets need FbpA, B or C
	*gltA*	Glutamate synthase (iron-sulfur)		
	*lutABC*	Iron-sulfur oxidase		
	*dctP*	Dicarboxylate permease		
	*leuCD*	Leucin biosynthesis		
RoxS (115)	*ppnKB, sucC, yflS*	Redox regulation, TCA cycle	TI, RD	ResD (NO), Rex (malate)
RnaC (125?)	*abrB*	Transition state	RD?,TI?	Growth phase

**TI, translational inhibition; RD, promotion of mRNA degradation; ?, proposed but not experimentally shown. For further details see text.**

**SR1** (205 nt) has been discovered using a computational search for sRNAs in intergenic regions of the *B. subtilis* genome and later confirmed by Northern blotting (Licht et al., 2005). Its transcription is repressed under glycolytic conditions ≈20–30-Fold by CcpN binding upstream of and overlapping the *sr1* promoter p*_*sr*__1_* and, to a minor extent, by CcpA binding to a site ∼260 bp upstream of the transcription start site. CcpN needs ATP and a slightly acidic pH to exert its effect (Licht et al., 2008) and interacts with the α-subunit of the RNA polymerase to prevent promoter escape (Licht and Brantl, 2009). The first identified primary target of SR1 is *ahrC* mRNA (Heidrich et al., 2006) encoding the transcription activator of the arginine catabolic operons *rocABC* and *rocDEF* and the transcription repressor of the arginine biosynthesis genes (Czaplewski et al., 1992). In contrast to many other sRNAs, SR1 neither affects the half-life nor the amount of *ahrC* mRNA (Heidrich et al., 2006). Binding of SR1 and *ahrC* mRNA via their 7 complementary regions (A to G in SR1, A′ to G′ in *ahrC* mRNA) results in inhibition of *ahrC* translation initiation by a novel mechanism: induction of structural changes 20 to 40 nt downstream of the RBS, although SR1 binds ∼100 nt downstream of the *ahrC* RBS (Heidrich et al., 2007; Figure 3A). The interaction between SR1 and *ahrC* mRNA initiates at region G/G′ and is the crucial interaction, but the other complementary regions also contribute to complex formation and hence, translation inhibition (Heidrich et al., 2007). Recently, it was shown that the abundant RNA chaperone CsrA promotes the interaction between SR1 and *ahrC* mRNA: It binds both RNAs and induces a slight structural alteration in *ahrC* mRNA which liberates region G’ for a more efficient interaction with SR1 (Müller et al., 2019; Figure 3A).

**FIGURE 4:**
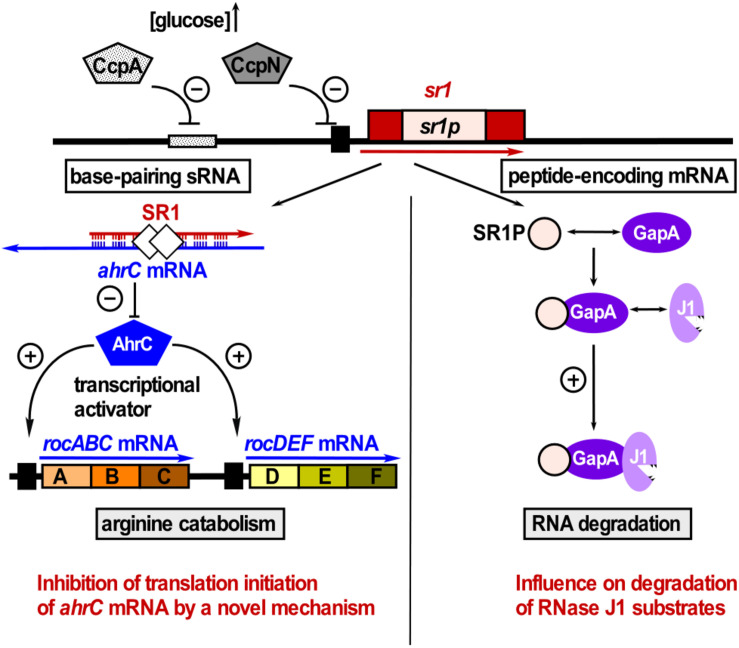
SR1 is a dual-function sRNA. Left: SR1 acts as base-pairing sRNA in arginine catabolism by inhibiting translation of *ahrC* mRNA encoding the transcription activator of the arginine catabolic operons. SR1 transcription is inhibited by repressors CcpA and CcpN. CsrA (white diamonds) promotes the SR1-*ahrC* mRNA interaction (Müller et al., 2019). Right: SR1 is an mRNA encoding SR1P that plays a role in RNA degradation. SR1P interacts with GapA thereby promoting the GapA-RNase J1 interaction and the enzymatic activity of RNase J1 (adapted with permission from Gimpel and Brantl, 2017).

SR1 is not only a *trans*-encoded base-pairing sRNA, but also an mRNA encoding a 39 aa protein, SR1P (Gimpel et al., 2010). SR1P interacts with the glyceraldehyde-3P dehydrogenase GapA, thereby affecting RNA degradation (Gimpel and Brantl, 2016, 2017, see below and Figure 4). Both functions of SR1 are remarkably conserved over more than one billion years of evolution (Gimpel et al., 2012).

In 2008, **FsrA** (84 nt), a functional homolog of *E. coli* RyhB, was discovered (Gaballa et al., 2008). FsrA is under transcriptional control of the iron-dependent Fur repressor. When iron is scarce, FsrA inhibits translation of target mRNAs involved in iron metabolism and storage, e.g., succinate dehydrogenase *sdhCAB* (Figure 3B), *citB* (aconitase) and *lutABC* (oxidases important for growth on lactate as sole C source). Subsequent studies revealed that FsrA is a global regulator with a broad variety of targets, among them *gltAB* encoding the iron-sulfur containing enzyme glutamate synthase, *dctP* (dicarboxylate transporter), *resA* and *qcrA.* By predicting complementary regions with RNAhybrid and M-fold the authors suggest that a C-rich single-stranded region of FsrA base-pairs with the RBS of its target mRNAs (Smaldone et al., 2012a). So far, *in vivo* base-pairing has only been confirmed between FsrA and the 5′ UTR of *gltAB* (Smaldone et al., 2012a; Figure 3B). *In vitro* base-pairing was demonstrated by EMSA for FsrA and *sdhC* mRNA, whereas for the other targets, only effects on mRNA and protein levels were reported (Gaballa et al., 2008). In contrast to the functionally related Fur-regulated sRNA RyhB from *E. coli*, which requires the RNA chaperone Hfq, FsrA cooperates with one, two or three Fur-regulated small basic proteins FbpA, FbpB, and FbpC suggested to be RNA chaperones. Under iron-deplete growth conditions, FsrA and FbpB (48 aa) inhibit *lutABC* to allow the direction of iron to higher-priority target proteins. Both target mRNA and protein levels were affected about two-fold by FsrA and FbpB with FbpB having a stronger effect on RNA levels. Whereas FsrA might directly inhibit translation, FbpB might facilitate FsrA/*lutABC* RNA pairing or recruit the RNA degradation machinery (Smaldone et al., 2012b). Currently, there is neither experimental evidence for FbpB binding RNA nor for its mechanism of action. Neither for *sdhCAB* mRNA nor for *citB* mRNA a contribution of one of the Fbp proteins to FsrA-mediated repression was found (Gaballa et al., 2008).

**RoxS** (originally termed RsaE, 115 nt) is the only base-pairing sRNA conserved between *B. subtilis* and *S. aureus.* RoxS transcription is activated by nitric oxide depending on the two-component system ResDE (Durand et al., 2015) and repressed by the NADH sensitive Rex (Durand et al., 2017). Repression is released by malate. RoxS helps to restore the NAD^+^/NADH balance by temporarily turning down part of the TCA cycle. Experimentally confirmed direct RoxS targets are *ppnKB* mRNA (NAD^+^/NADH kinase), *sucCD* mRNA (succinyl-CoA synthase) and *yflS* (one of four malate transporters; Durand et al., 2015, 2017). RoxS inhibits translation and promotes degradation of *ppnKB* and *sucC* mRNAs (Figure 3C). RNase III cleaves the RoxS/*ppnKB* RNA duplex and RNase Y cleaves *ppnK* mRNA both RoxS-dependently but also -independently. For translation regulation, one of four CCC rich regions of RoxS (CCR3) suffices. RoxS itself is also subject of RNase Y cleavage at nt + 20 yielding truncated RoxS(Y) which can efficiently regulate *ppnKB* and is required for translation inhibition of *sucCD* operon RNA. In contrast to *ppnKB* and *sucCD*, *yflS* is positively regulated: RoxS activates *yflS* translation by disrupting a structure at the 5′ UTR that impedes ribosome recruitment. In addition, RoxS stabilizes *yflS* mRNA by preventing 5′-3′ exonucleolytic degradation by RNase J1 (Figure 3C). Both effects are independent and require RoxS region CCR3. Hfq is not involved in RoxS-dependent regulation of *ppnKB* mRNA (Durand et al., 2015).

**RNAC/S1022** was first identified in a microarray screen of *B. subtilis* intergenic regions, but has so far not been detected in Northern blots. It is transcribed exclusively under control of *σ*^D^ during logarithmic growth in LB medium (Schmalisch et al., 2010). RNAC modulates the cellular level of transition state regulator AbrB via base-pairing with *abrB* mRNA at the RBS and the first six codons (Mars et al., 2015b; Figure 3D). The Hfq-independent RNAC/*abrB* RNA interaction seems to promote degradation of *abrB* mRNA and might also inhibit its translation. However, only minimal alterations (≈33%) of mRNA and protein levels were observed in Δ*rnaC* strains. RNAC increases the cell-to-cell variation of the AbrB levels, which leads to growth rate heterogeneity of cells within one population during exponential phase. This heterogeneity is physiologically relevant, since slowly growing bacterial cells are less susceptible to antibiotics and environmental stress (Mars et al., 2015b).

### The T-box Riboswitch

Another example of regulatory RNA-RNA interactions is the T-box riboswitch, first discovered in the *B. subtilis tyrS* gene (Henkin et al., 1992). It is an example for transcription attenuation mechanisms that are based on the ability of an mRNA leader region to fold into two mutually exclusive structures, a transcription terminator or an antiterminator, depending on ribosome movement, binding of proteins, an antisense RNA, small ligands or – in the T-box– the loading state of a tRNA (rev. in Brantl, 2004).

The T-box riboswitch is mainly found in Firmicutes to control the expression of amino acid related genes (aa biosynthesis or transport, aminoacyl-tRNA synthetases). Grundy et al. (1994) showed that a UAC to UUC mutation in the *tyrS* 5′ UTR suffices to induce expression under phenylalanine instead of tyrosine limiting conditions indicating that a tRNA was involved in regulation.

**FIGURE 5:**
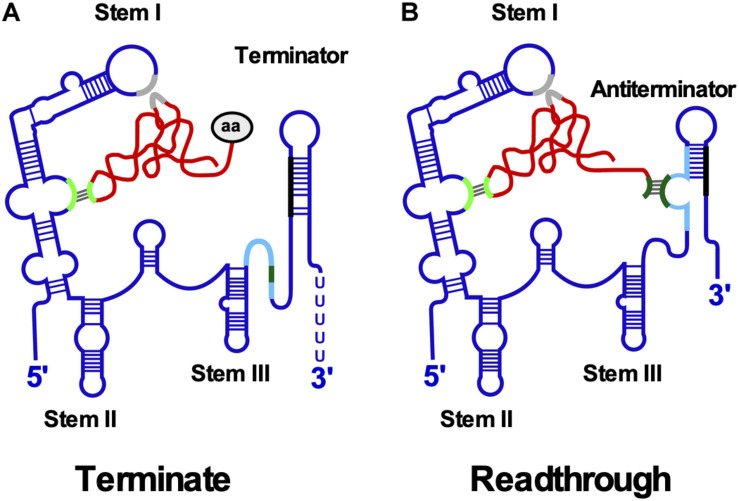
T-box riboswitch. **(A)** Interaction between T-box leader RNA and tRNA occurs at two positions. Aminoacylated tRNA binds the specifier loop (light green) in stem I through base-pairing and the stem I platform (gray) by stacking interactions. The aa at the 3′ end prevents binding of the acceptor arm to the terminator helix. The more stable terminator helix (black-blue) forms and transcription terminates. **(B)** Uncharged tRNA interacts with specifier loop and stem I platform. The free acceptor arm of tRNA (dark green) binds the antiterminator (light blue) and stabilizes it allowing transcription to read through the termination site into the downstream gene. Conserved structural domains including stems I-III and the mutually exclusive terminator and antiterminator helices are labeled. The tRNA is shown in red and the aa in gray.

T-box leader RNAs are composed of stems I, II and III and a pseudoknot (Stem IIA/B) present upstream of the competing terminator and antiterminator helices (Figure 5). Stem I harbors an internal loop with the specifier sequence that base-pairs with the tRNA anticodon. The stem I distal region comprises conserved sequence motifs and a terminal loop which interact to form a contact surface with the tRNA D-/T-loops through stacking interactions (Grigg and Ke, 2013; Zhang and Ferré-D’Amaré, 2013). Both an E-loop and a pseudoknot directly downstream of size-variable stem II are required for efficient antitermination. Stem III upstream of the antiterminator varies significantly in sequence and length (rev. in Kreuzer and Henkin, 2018).

Downstream gene expression depends on the ratio between charged and uncharged tRNAs (Figure 5): Both tRNAs bind to the specifier loop and stem I platform. However, the presence of the charged tRNA aa prevents the interaction of the acceptor end with the antiterminator bulge, facilitating formation of a more stable terminator helix causing premature transcription termination. By contrast, the acceptor arm of uncharged tRNA base-pairs with the antiterminator bulge thereby stabilizing the antiterminator, allowing read-through into the downstream ORF. Binding of uncharged tRNA induces structural changes throughout the leader RNA. An smFRET analysis of *B. subtilis glyQS* (Zhang et al., 2018) demonstrated a two-step T-box-tRNA interaction comprising the interaction with the anticodon followed by sensing of the 3′ NCCA end. The association rate constant for the first binding step was with 5.0 × 10^5^ M^–1^ s^–1^ comparable to that of *cis*-encoded antisense RNAs (see above).

A cryo-EM structure of the *B. subtilis* T-box riboswitch-tRNA complex (Li et al., 2019) revealed a 66 nt functional unit – T-box discriminator – that selectively binds uncharged tRNA. It is formed of stem III with flanking purines and the adjacent antiterminator. The T-box adopts a U-shaped molecular vice that clamps the tRNA. The discriminator captures the tRNA 3′ end with nanomolar affinity and uses a steric filter fashioned from a G-U wobble bp to determine its aminoacylation state. When the tRNA is uncharged, the T-box clutches it and forms a continuously stacked central spine allowing transcriptional readthrough.

## RNA-Protein Interaction

### RNA Chaperones

RNA chaperones are proteins binding to RNAs and affecting their structure or stability and therefore influencing both translation of mRNAs and regulatory functions of sRNAs. *B. subtilis* encodes two major RNA chaperones: Hfq and CsrA, which are both well investigated in Gram-negative bacteria (rev. in Vakulskas et al., 2015; Kavita et al., 2018) and have a broad impact on gene regulation.

### Hfq

As in other Gram-positive bacteria, the role for Hfq in *B. subtilis* seems to be minor. Only around 150 transcripts could be identified to bind Hfq and even less were affected in their abundance (Dambach and Winkler, 2013; Hämmerle et al., 2014). The structure of *B. subtilis* Hfq is similar to that of *E. coli* Hfq, but it prefers binding to AG-repeats instead of ARN-repeats (Someya et al., 2012). So far, no case has been described where *B. subtilis* Hfq facilitates the interaction between two RNAs, a major task of *E. coli* and *Salmonella* Hfq with numerous examples and a pleiotropic phenotype in its absence. In contrast, in a huge screen with more than 2000 growth conditions no growth differences between a wild-type and an isogenic *hfq*-deficient *B. subtilis* strain were detected (Rochat et al., 2015). The only known phenotypes in the absence of *hfq* are a decreased long-term survival of cells in stationary phase, independent of sporulation (Rochat et al., 2015) and a drastically impaired motility and chemotaxis (Jagtap et al., 2016).

Remarkably some toxin mRNAs as well as the corresponding antitoxins of type I TA systems are bound by Hfq (Dambach and Winkler, 2013) and are less abundant in its absence (Hämmerle et al., 2014). However, this had no impact on growth or survival (Rochat et al., 2015). Similarly, Hfq had no effect on the type I TA system *bsrE*/SR5, although it stabilizes SR5 (Müller et al., 2016). The expression of *hfq* is upregulated in stationary phase and under several stress conditions (Dambach and Winkler, 2013; Rochat et al., 2015; Jagtap et al., 2016). Together this rather implies a role for Hfq as a general stationary phase fine-tuning regulator than as a globally acting RNA-RNA matchmaker in *B. subtilis.* Interestingly Hfq binds *in vitro* near the SD sequence of *ahrC* mRNA, refolds the 5′ UTR to allow access to the 30S SU and is required for *ahrC* translation *in vivo*, but dispensable for its inhibition by SR1 (Heidrich et al., 2007, see above). This confirms that Hfq is a chaperone in *B. subtilis*, but its role may be restricted to mRNAs.

### CsrA

The second RNA chaperone, CsrA, also differs from its *E. coli* relative. Despite the highly similar general structure of the *B. subtilis* CsrA dimer (Altegoer et al., 2016) as well as the shared affinity for GGA motifs in single-stranded and looped-out RNA structures, only one major function in motility regulation in *B. subtilis* was described in detail (Yakhnin et al., 2007): The major flagellin protein Hag, the regulatory protein FliW, CsrA and the *hag* mRNA form a regulatory circuit to limit the intracellular Hag concentration. At high concentrations, Hag binds FliW, and CsrA is free to block Hag synthesis by *hag* mRNA binding, otherwise FliW sequesters CsrA and *hag* is expressed (Mukherjee et al., 2011). Nevertheless, the interaction with FliW does not exclude RNA binding by CsrA in general (Altegoer et al., 2016; Mukherjee et al., 2016), and further functions of CsrA are conceivable.

Only recently, a novel mechanism was discovered, where CsrA refolds the *ahrC* mRNA to facilitate binding of the regulatory sRNA SR1 (Müller et al., 2019, Figure 3A). This also raises the question of a more global role of CsrA in *B. subtilis*.

### tmRNA

*Trans*-translation is an omnipresent bacterial mechanism to rescue ribosome stalling on mRNAs without stop-codon, to tag the nascent peptide and to initiate its degradation (rev. in Himeno et al., 2014, 2015). The functional complex comprises the tmRNA (*ssrA*) and the protein SmpB (*ssrB*). Together the complex mimics the structure of tRNA^Ala^, is bound by EF-Tu-GTP and can be loaded onto stalled ribosomes. The globular domain of SmpB acts as a substitute of the anticodon-loop and occupies the decoding site, while the C-terminal tail of SmpB probes the vacant mRNA channel of the 30S SU (rev. in Miller and Buskirk, 2014). In contrast to normal tRNAs, the tmRNA has neither an anticodon-loop nor a D-loop. SmpB compensates for these structures and allows recognition by the ribosome and also fine-tunes the tmRNA structure (Miller and Buskirk, 2014). An additional looped out coding strand of tmRNA is used by the ribosome as mRNA template that encodes the species-specific tag sequence, in *B. subtilis* (A)GKTNSFNQNVALAA. Finally, the ribosome is released and the truncated and tagged protein degraded by Clp proteases that recognize the ALAA-motif (Wiegert and Schumann, 2001; Kolkman and Ferrari, 2004, Figure 6).

**FIGURE 6:**
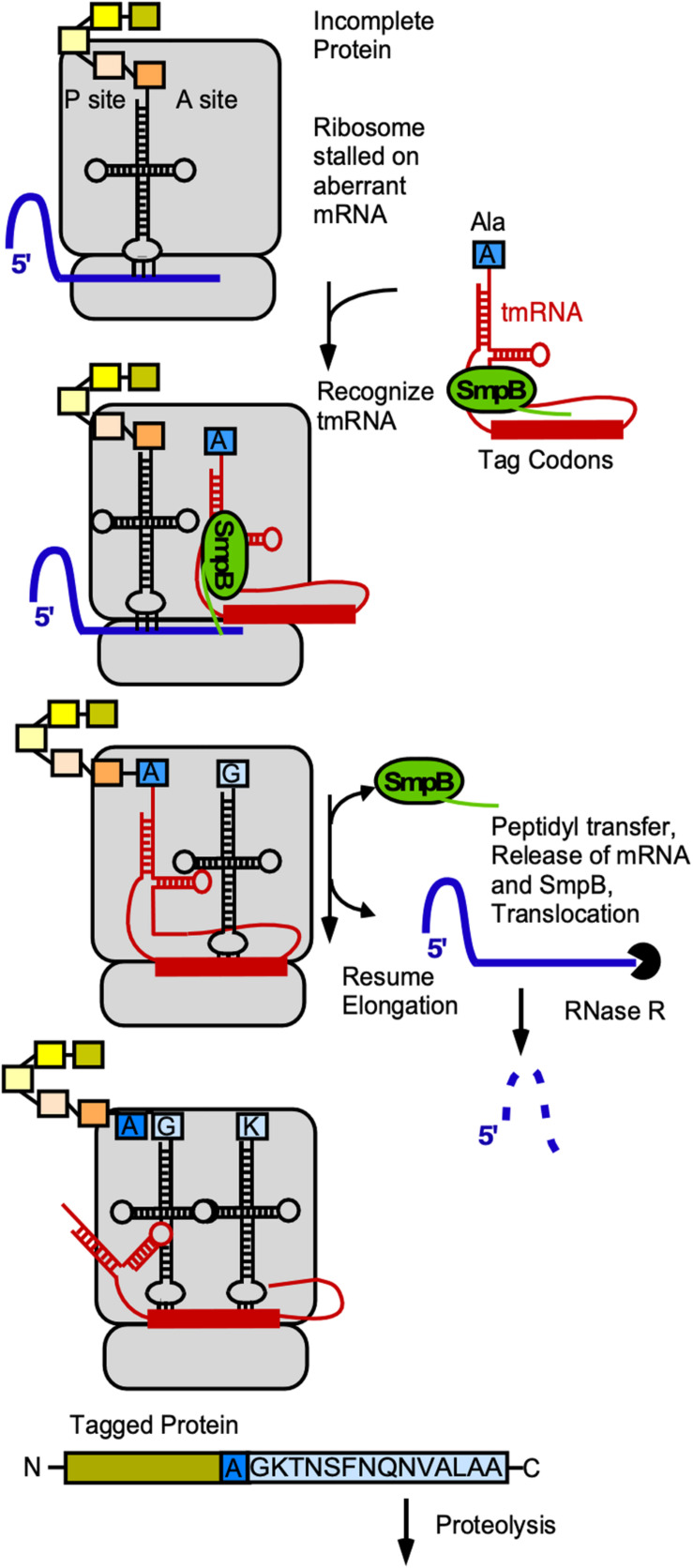
Role of tmRNA and SmpB in *trans*-translation. Gray, ribosome; blue, mRNA, red, tmRNA; yellow/orange-colored rectangles, aa in peptide chain associated with last tRNA; blue rectangle, alanine with which tRNA was charged by alanyl-tRNA synthetase; light-blue, tag encoded by tmRNA. For details see text.

*Trans*-translation is important under specific stress conditions to maintain error-free gene expression and rescue stalled ribosomes. In *B. subtilis* both *ssrA* and *ssrB* are important for growth at temperature extremes and under ethanol and heavy metal ion stress (Muto et al., 2000; Shin and Price, 2007). They are encoded in one operon – together with the gene for RNase R that degrades the aberrant mRNA – and upregulated under stress conditions by a *σ*^B^-dependent promoter (Muto et al., 2000).

Surprisingly, *trans*-translation is not only involved in the prevention of defective expression under stress conditions, but also part of gene regulation in general (rev. in Keiler, 2015). In *B. subtilis*, the system is essential in the absence of a second ribosome rescue system, BrfA/PrfB, which uses a distinct mechanism and is directly downregulated by *ssrA-smpB*-dependent *trans*-translation (Shimokawa-Chiba et al., 2019). The expression of several genes depends on the tmRNA-SmpB-mechanism (Fujihara et al., 2002). It is also involved in carbon catabolite repression: CcpA binding to *cre*-sites causes a stall of transcription and subsequently translation, which is finally solved by *trans*-translation (Ujiie et al., 2008). Furthermore, *trans*-translation is important for efficient sporulation: In Δ*ssrA* cells, the formation of *sigK –* coding for a sporulation dependent sigma factor – by a DNA rearrangement between *spoIIIC* and *spoIVCB* is impaired, leading to drastically reduced sporulation efficiency (Abe et al., 2008). It is also likely that other physiological processes could be affected by *trans*-translation in general.

### 6S RNA

**FIGURE 7:**
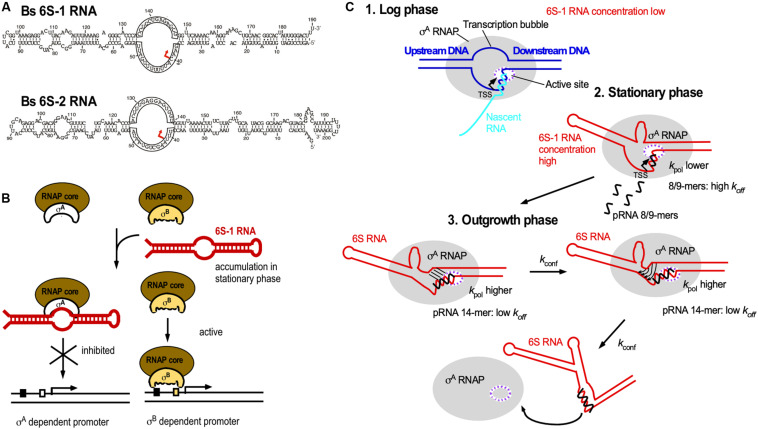
6S RNA. **(A)** *B. subtilis* encodes two 6S RNAs, 6S-1 and 6S-2. Red arrow, TSS for pRNAs. **(B)** Biological function of 6S RNA. By mimicking an open promoter, 6S-1 RNA, which is abundant in stationary phase, can sequester *σ*^A^-RNAP thereby preventing transcription initiation at *σ*^A^-dependent promoters. *σ*^B^-RNAP is not bound by 6S-1 RNA, thus allowing transcription from *σ*^B^-dependent promoters. **(C)** Mechanism of action of pRNAs. Black, pRNA; *k*_*pol*_, rate constant of polymerization (synthesis) of pRNAs; *k*_*off*_, dissociation rate constant of pRNAs; *k*_*conf*_, rate constant for refolding of 6S RNA. The ratio between *k*_*pol*_, *k*_*off*_, and *k*_*conf*_ determines whether or not pRNAs dissociate or 6S-1 RNA refolds. For detailed explanations see text. **(C)** is modified based on Beckmann et al., 2012, with permission.

The ≈200 nt long 6S RNA is ubiquitous in bacteria, and *B. subtilis* has even two 6S RNAs, 6S-1 and 6S-2. All 6S RNAs have a characteristic secondary structure with a central single-stranded loop flanked by two irregular double-stranded stems, which are interrupted by small bulges (Figure 7A). In 2000, it was discovered that 6S RNA forms a stable complex with *E. coli* RNA polymerase (RNAP) to regulate its activity (Wassarman and Storz, 2000). It interacts with the RNAP β/B’ subunit and *σ*^70^, but not with the stationary phase *σ*^S^. Therefore, it can in stationary phase, when its amount increases to 10.000 molecules/cell, repress transcription at vegetative promoters (Figure 7B). Crystal structure analysis revealed that *E. coli* 6S RNA mimics B-form DNA (Chen et al., 2017) allowing it to mimic an open promoter thus interfering with the formation of transcription initiation complexes (Figure 7B). 6S RNA acts as template for the synthesis of 14–22 nt pRNAs that are required to relieve 6S-dependent transcription inhibition and thus, recovery of cells from stationary phase in *B. subtilis* and *E. coli* (Beckmann et al., 2011; Cavanagh et al., 2012). Both *B. subtilis* 6S-1 and 6S-2 are *in vitro* and *in vivo* templates for pRNA synthesis (Burenina et al., 2014; Hoch et al., 2016). 6S-1 is highest expressed in stationary phase, allows growth adaptation and prevents premature sporulation (Cavanagh and Wassarman, 2013) whereas 6S-2 is more abundant in logarithmic phase, and its function is still unknown. In 2012, the mechanism of action of *B. subtilis* 6S-1 RNA was elucidated (Beckmann et al., 2012; Figure 7C): As soon as the newly synthesized pRNA has formed a sufficiently stable duplex with 6S-1 RNA, it induces *in cis* a refolding of the 6S RNA that involves a base-pairing between the 5′ part of the central bulge and nucleotides that are available due to pRNA invasion. This rearrangement decreases the affinity of 6S-1 to the RNAP. Only 12–14 nt long pRNAs, but not shorter ones, are stable enough to induce a refolding. The ratio between the rate constants for polymerization (k_*pol*_), pRNA:6S RNA-dissociation (k_*off*_) and refolding (k_*conf*_) determines whether or not pRNAs dissociate or refold the 6S-1 RNA.

Future investigations will reveal the function of 6S-2 and show which mechanisms mediate promoter-specific transcription regulation by both 6S RNAs.

### RNA Binding Proteins Inducing Terminator or Antiterminator Formation

Examples for transcription attenuation (see above and Figure 5) depending on protein binding involve *B. subtilis* TRAP (*trp* RNA binding attenuation protein) and various antitermination proteins.

**FIGURE 8:**
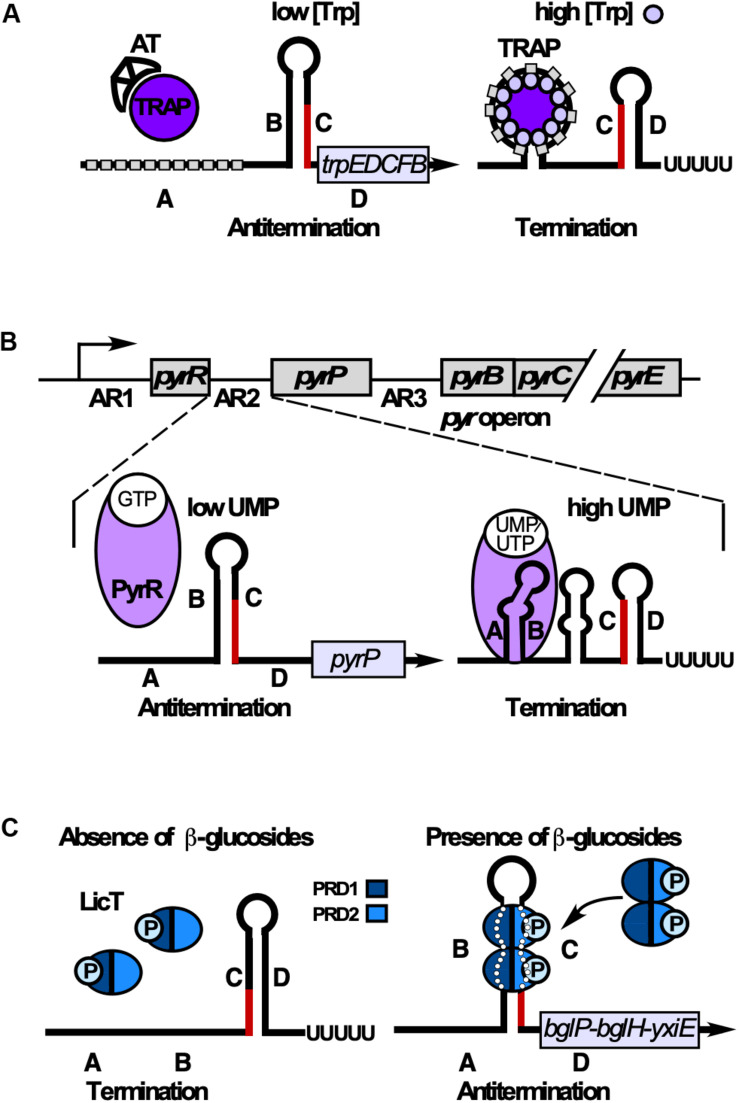
RNA-binding proteins that induce termination or antitermination. Alternative folding occurs by base-pairing between regions B and C (antiterminator) or C and D (terminator). B and C overlap**. (A)** Control of tryptophan (Trp) biosynthesis. When Trp is scarce, anti-TRAP (AT) is expressed and sequesters TRAP. Regions B and C base-pair preventing terminator formation. At excess Trp, 11-mer TRAP binds Trp enabling each subunit to bind one G(U)AG triplet (gray rectangle) through contacts with aa K37, K56, and R58. This results in RNA wrapping around TRAP in regions A and B. Therefore, B cannot base-pair with C, allowing terminator formation. **(B)** Top: The 5′ end of the *pyr* operon mRNA contains 3 attenuation regions (AR1-3) upstream of the *pyrR*, the *pyrP* and the *pyrB* ORF, respectively. Bottom: Control of UMP synthesis. At low UMP/UTP but high GMP levels, PyrR binds GTP and is unable to bind RNA. B and C base-pair preventing formation of the terminator and thus, termination of the *pyr* operon. At high UMP/UTP levels, PyrR binds UMP or UTP, but not GTP and stabilizes the A-B antianti-terminator, thus preventing of B-C-antiterminator formation. Instead, the terminator stem-loop forms resulting in premature transcription termination. **(C)** Control of utilization of β-glucans/β-glucosides. In the absence of salicin (β-glucoside), PRD1 is phosphorylated by BglP, causing LicT inactivation (monomers). In the presence of salicin, BglP dephosphorylates PRD1 and transfers the phosphate to incoming salicin, and HPr phosphorylates PRD2. This allows LicT to dimerize and bind/stabilize the otherwise unstable antiterminator.

**TRAP** (Babitzke and Yanofsky, 1993; Otridge and Gollnick, 1993) is composed of 11 subunits stabilized through 11 intersubunit β-sheets to form a β-wheel with a large central hole of 80 Å diameter (Antson et al., 1999). L-tryptophan binding in clefts between adjacent β-sheets induces conformational changes. The unstructured *trp* operon 5′ UTR contains 11 U/GAG repeats spaced by 2–3 nt and forms a matching circle around Trp-bound TRAP using mostly specific protein-base interactions (Figure 8A) to cause transcription termination (rev. in Babitzke et al., 2019). When tryptophan is scarce, uncharged *^trp^*tRNA allows expression of Anti-TRAP that binds TRAP to inhibit its activity. The RNA refolds into an antiterminator and transcription of *trpEDCFBA* continues (rev. in Babitzke et al., 2019; Figure 8A). Interestingly, TRAP-mediated termination is neither intrinsic nor Rho-dependent. Instead, a region around aa E60 which is not involved in Trp or RNA binding (McAdams et al., 2017) allows TRAP to induce forward translocation of the RNAP, which is required for efficient termination (Potter et al., 2011). Both the *trpE* and *trpG* genes are regulated by TRAP exclusively at translational level (rev. in Babitzke et al., 2019).

The *pyr* operon contains 10 genes encoding all enzymes for UMP *de novo* synthesis under control of one constitutive promoter. **PyrR**, one of the two *B. subtilis* uracil-phosphoribosyltransferases, moonlights as RNA binding attenuation protein in regulation of this operon in three identical attenuation mechanisms (Turner et al., 1994, Figure 8B). An UMP-bound PyrR dimer stabilizes the anti-antiterminators comprising ARUCCAGAGAGGYU to allow formation of downstream terminators (rev. in Turnbough and Switzer, 2008; Turnbough, 2019). PyrR recognizes only conserved RNA sequences that are properly positioned in the correct secondary structure: terminal loop, top of the upper stem and a purine-rich internal bulge are crucial for efficient PyrR binding (Bonner et al., 2001) which involves a basic concave (Savacool and Switzer, 2002). The crystal structure of *B. caldolyticus* PyrR, which is active in *B. subtilis pyr* operon regulation (Chander et al., 2005) revealed an unexpected specific GMP binding antagonistic to RNA binding which suggests cross-regulation of the *pyr* operon by purines. Interestingly, in each of the three attenuation sites, NusA stabilizes *in vitro* pausing of the RNA polymerase at a major pause site to prevent formation of a complete antiterminator thus promoting formation of a PyrR binding loop (Zhang and Switzer, 2003). This might also play a role in termination of *pyr* transcription *in vivo*.

Antitermination proteins **GlcT, SacY, SacT**, and **LicT** contain an N-terminal RNA binding domain and regulatory domains PRD1 and PRD2 which are reversibly phosphorylated in response to the cognate carbon source (rev. in Stülke, 2002). In the absence of the carbon source, the cognate EII transporter of the phosphotransferase system phosphorylates PRD1 preventing dimerization. In its presence, EII dephosphorylates PRD1, while HPr phosphorylates PRD2, which allows dimerization and antiterminator binding (Figure 8C).

Crystal structures of inactive LicT revealed a wide swing movement of PRD2 causing a dimer opening that brings the phosphorylation sites to the protein surface (Graille et al., 2005). LicT interacts with two bulges in the antiterminator and the minor groove of the stem between them (Yang et al., 2002, Figure 8C). In GlcT, arrangement of the PRDs is under selective pressure to ensure a proper regulatory output (Himmel et al., 2012). For SacT, SacY, and LicT, specificity domains were identified that prevent a cross-talk between these systems (Hübner et al., 2011).

Glycerol-3P-bound **GlpP** binds the antiterminator and additionally stabilizes *glpD* mRNA (rev. in Stülke, 2002). Hexameric HutP (histidine utilization protein) prevents terminator formation in the *hut* operon. Coordination of L-histidine and Mg^2+^ activates HutP to bind two clusters of three NAG repeats spaced by 20 nt without undergoing further structural rearrangements (rev. in Kumarevel, 2007).

### Aconitase CitB, a Metabolic Enzyme That Moonlights as RNA Binding Protein

**FIGURE 9:**
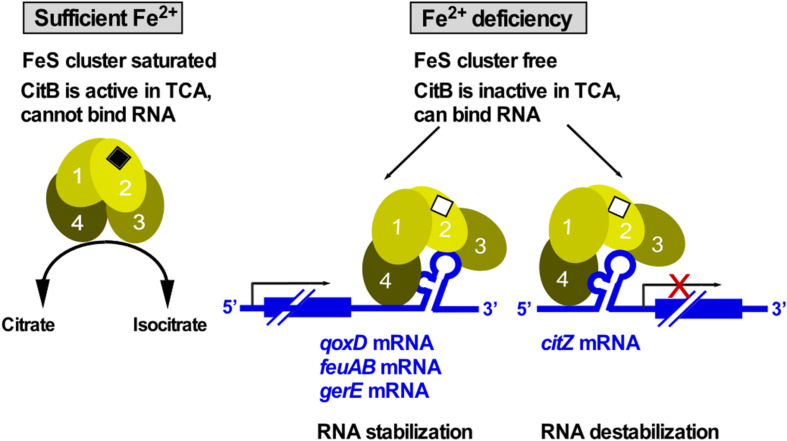
CitB – a dual-function enzyme that moonlights in RNA binding. Under iron-rich conditions, the FeS cluster of CitB is saturated allowing it to function as metabolic enzyme in the TCA. Under iron limitation, the FeS cluster is unsaturated resulting in a conformational change that allows CitB to bind RNA at iron-response elements (IREs) or stem-loops in 3′ UTRs to stabilize the corresponding RNAs or at the 5′ UTR to destabilize the RNA. Depicted conformational changes and domain numbers are based on the crystal structure of the human iron regulatory protein 1 complexed with ferritin IRE-RNA (see text, Walden et al., 2006).

Aconitase is an enzyme that operates in the tricarboxylic acid cycle (TCA) of all three kingdoms of life to convert citrate into isocitrate. For this activity, it needs a saturated iron-sulfur (FeS) cluster. Under iron limitation, the FeS cluster disassembles causing enzyme inactivation and, therefore, TCA shutdown. The inactive enzyme adopts an alternative conformation to bind at iron-response elements (IREs) located in the 5′ or 3′ UTR of mRNAs to repress translation or RNA degradation, respectively. The first bacterial aconitase discovered to bind RNA was *B. subtilis* CitB (Alén and Sonnenshein, 1999): Monomeric CitB binds *in vitro* at an IRE in the 3′ UTR of *qoxD* (cytochrome oxidase subunit) and another IRE between *feuA* and *feuB* (iron uptake). In addition, it binds at a stem-loop in the 3′ UTR of *gerE* mRNA encoding a transcription factor in sporulation, which might stabilize the RNA to ensure proper timing of spore coat formation (Serio et al., 2006). However, half-life measurements of *qoxD, feuAB* or *gerE* mRNAs have not yet been performed to confirm stabilization of these RNAs by CitB under iron limitation. Binding of CitB to the 5′ UTR of *citZ* mRNA resulted in destabilization of the three transcripts originating at the *citZ* promoter which is in line with prevention of translation (Pechter et al., 2013; Figure 9). Surprisingly, CitB bound *in vitro* both *citZ* mRNA and the negative control *hag* mRNA. To date, only the structure of the dual function human iron-regulatory protein 1 in complex with ferritin RNA has been solved (Walden et al., 2006). Under Fe^2+^ limitation, aconitase adopts a more open conformation with structural domains 3 and 4 extending perpendicularly from the central core composed of domains 1 and 2 (Figure 9). Direct contacts between the exposed IRE loop residues AGU and the domain 2/domain 3 interface provide specificity and stability to the interaction (Walden et al., 2006). Protein binding to the lower stem of the IRE is centered on a – sometimes bulged out – C nucleotide, which inserts into a pocket on the inner face of domain 4. Due to the high conservation of aconitase, similar structural changes most likely occur in *B. subtilis* CitB as well (Figure 9), although only in the case of *feuAB*, the IRE loop comprises an AGU motif.

## Small Protein-Protein Interactions

**FIGURE 10:**
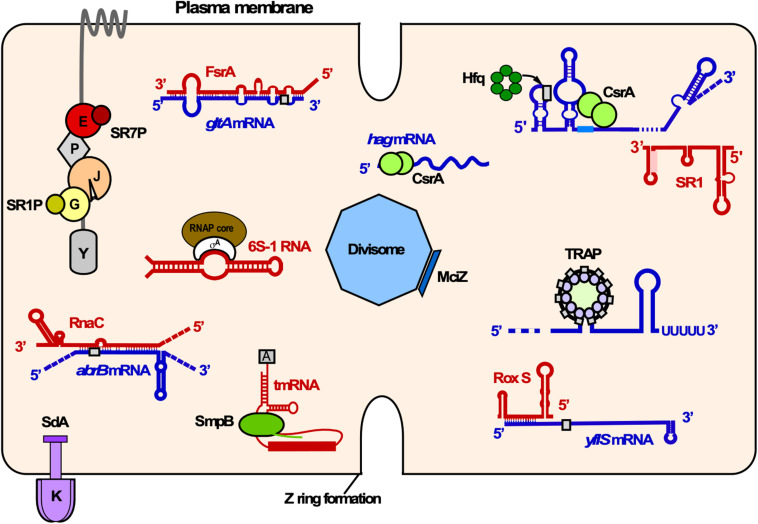
Overview of RNA-RNA, RNA-protein and small protein-protein interactions in *B. subtilis*. Shown is the cytosol of *B. subtilis* bounded by the plasma membrane. Proteins associated with various cellular functions are labeled as follows: K, KinA; E, enolase; P, PfkA; J, RNase J1; G, GapA. Small proteins are depicted as rectangles or solid circles. Only components of the degradosome mentioned in the text are illustrated. Red, sRNAs and other regulatory RNAs; blue, mRNAs; gray rectangles, RBS; different shades of green, RNA binding proteins. Other colors, protein-small protein interactions. Y, RNase Y.

Small proteins encoded by small ORFs comprise less than 50 aa and are involved in the regulation of a number of cellular functions including morphogenesis, cell division, enzymatic activities and stress response (rev. in Storz et al., 2014). Until recently, small ORFs had escaped the researchers’ attention as their detection was difficult. With the advent of new technologies, thousands of translated small ORFs have been recently identified in prokaryotes and eukaryotes. However, the identification and characterization of small proteins is still challenging. Here, we review *B. subtilis* small proteins that interact with larger proteins (Figure 10).

### *Sda* Interacts With KinA and Affects Signal Transduction

An example for small proteins localized at the inner membrane that interact with transmembrane sensor kinases is the 46 aa Sda. It binds to and inhibits KinA, the first histidine kinase in the phosphorelay that regulates *B. subtilis* sporulation. Upon starvation and stress, kinases KinA and KinB autophosphorylate and transfer their phosphates via Spo0F and Spo0B to the central regulator Spo0A. The *sda* gene was identified in a *dnaA1* suppressor mutant which was able to sporulate. Its promoter region contains multiple DnaA binding sites, and mutations in these regions or in *dnaA* affected *sda* expression (Burkholder et al., 2001). Structural studies showed that the Sda monomer is composed of two α-helices held together by an interhelix loop forming a helical hairpin (Rowland et al., 2004). The Sda-KinA interaction surface was mapped: Sda uses a hydrophobic surface portion formed by L21 and F25 to interact with the KinA dimerization/phosphotransfer (DHp) domain (Rowland et al., 2004). Several mechanisms for KinA inhibition by Sda were proposed: Sda might prevent KinA autophosphorylation by interrupting the phosphate transfer between the ATP binding site in the catalytic and the DHp domain (Rowland et al., 2004). Sda could also induce conformational changes in the DHp domain which in turn interferes with the KinA-Spo0F-phosphotransfer (Whitten et al., 2007). A later study suggested that Sda directly blocks this phosphotransfer as its binding site on KinA overlaps with that of Spo0F (Cunningham and Burkholder, 2009). KinA regulation through Sda is advantageous for *B. subtilis*, as it contributes to modulate sporulation initiation at multiple levels in response to unfavorable conditions.

### MciZ Regulates Cell Division

Bacterial cytokinesis is initiated when the cell division machinery, the so called divisome, assembles at midcell. The divisome comprises about ten core proteins which help in cell membrane attachment and constriction (Lutkenhaus et al., 2012). Among them is FtsZ, which polymerizes into the Z ring in a GTP-dependent manner and exerts the actual constriction force (Shapiro et al., 2009). A number of small proteins interacting with FtsZ or other divisome components were identified. They also include MciZ (mother cell inhibitor of FtsZ) which was discovered in 2008 in a yeast two-hybrid screening using FtsZ as a bait protein (Handler et al., 2008). MciZ (40 aa) is produced under control of *σ*^E^ during sporulation (Handler et al., 2008). It impedes Z-ring formation by inhibiting FtsZ polymerization under moderate to low concentration. The crystal structure of the FtsZ-MciZ complex revealed a binding pocket for MciZ on one of the polymerization surfaces at the FtsZ C-terminus. MciZ binding inhibits FtsZ polymerization by steric hindrance (Bisson-Filho et al., 2015). Whereas the N-terminus of free MciZ is unstructured, the interaction with FtsZ induces the formation of two β-sheets (Bisson-Filho et al., 2015). At high concentration, MciZ sequesters FtsZ, while at sub-stoichiometric concentration, it acts by filament capping. Moreover, it can also cause filamentation *in vitro*. A recent study showed that MciZ can affect *B. subtilis* sporulation: Excessive amounts of MciZ produced intracellularly or added exogenously can not only decrease spore formation efficiency but also inhibit spore germination (Araújo-Bazán et al., 2019).

### Two Small Proteins Modulate the Degradosome

The proposed *B. subtilis* degradosome is composed of major endoribonuclease RNase Y, RNases J1 and J2, PNPase, helicase CshA, the glycolytic enzymes enolase and phosphofructokinase and under certain circumstances, GapA (Commichau et al., 2009; Gimpel and Brantl, 2016). So far, two small proteins – SR1P and SR7P – have been identified which interact with degradosome components.

**SR1P** is a 39 aa protein encoded by the dual-function sRNA SR1 (see above). It interacts with GapA, one of the two glyceraldehyde-3P-dehydrogenases (GAPDHs) in *B. subtilis.* SR1P stabilizes *gapA* mRNA by preventing its rapid degradation under gluconeogenesis (Gimpel et al., 2010). In addition to its role in glycolysis, GapA has a moonlighting property: It binds both RNases J1 and Y. SR1P promotes GapA binding to RNase J1 and enhances RNase J1 activity *in vitro* on at least two substrates, SR5 and threonyl-tRNA. In addition, it affects SR5 stability *in vivo* (Gimpel and Brantl, 2016). This means, SR1P modifies the moonlighting activity of GapA. Using peptide mutants in co-elution experiments complemented by SR1P functionality tests in Northern blotting, the SR1P-GapA interaction surface was determined. SR1P attaches to GapA using aa contacts in a binding pocket formed by the C-terminal GapA helix 14. In addition, SR1P contacts the N-terminal GapA helix 1 (Gimpel et al., 2017). Interestingly, SR1 is transcribed under gluconeogenic conditions, when GapB is active (Fillinger et al., 2000) and the glycolytic activity of GapA is not needed. Newman et al. proposed that RNA degradation is connected to the metabolic state of the cell (Newman et al., 2012). Similarly, it was hypothesized that under nutrient limitation, glycolytic enzymes like enolase, phosphofructokinase that are part of the *B. subtilis* degradosome as well as GapA might sense nutritional stress and transfer this signal to the RNA degradation machinery (Gimpel and Brantl, 2016, 2017). This could modulate the global RNA turnover rate to conserve energy for other important cellular functions.

**SR7P** (39 aa) previously known as S1136 (Mars et al., 2015a) is encoded by the dual-function antisense RNA SR7 (Ul Haq et al., 2020). The *sr7* gene is located in the intergenic region between *tyrS* and *rpsD* and controlled by a *σ*^B^-dependent promoter. SR7P is synthesized under five different stress conditions from the *σ*^B^-dependent SR7 as well as constitutively from a *tyrS* mRNA processing product. SR7P interacts with enolase present in the degradosome. This interaction in turn improves enolase binding to RNase Y. The SR7P-Eno-RNase Y interaction is not bridged by RNA. The activity of RNase Y is significantly higher in the tri-component SR7P/Eno/RNase Y complex than in the Eno/RNase Y complex alone. This modulating effect of SR7P was demonstrated both *in vivo* and *in vitro*. In addition, SR7P impacts cell survival under selective stress conditions suggesting that it might play a specific role in stress response.

## Conclusion and Perspectives

Although a variety of intermolecular interactions have been investigated in *B. subtilis*, major cavities in our knowledge still exist. Only for four of 108 *trans*-encoded sRNAs and for 8 small proteins, interaction partners have been identified and biological functions elucidated. Furthermore, a global approach is required to ascertain if CsrA plays a similar role in *B. subtilis* as Hfq and ProQ in Gram-negatives. The function of the putative RNA chaperones FbpA, FbpB and FbpC has to be unraveled. Targets of the multitude of sRNAs have to be identified and their – perhaps sometimes novel – mechanisms of action elucidated. A considerable increase in the number of dual-function sRNAs can be anticipated. sRNAs might be found that bind enzymes or act directly on the genome like some eukaryotic siRNAs. Structures of more antiterminator/protein complexes and CitB have to be solved and the function of 6S-2 RNA uncovered. The further study of small proteins which is still in its infancy, will allow insights into regulatory networks comprising not only small proteins modulating large proteins or the membrane but also those binding small ligands.

## Author Contributions

SB wrote the sections on RNA-RNA interactions, 6S RNA, terminators/antiterminators, CitB., and designed Figures 1–4, 6–9. IU wrote the sections on T-box riboswitch, small protein-protein interactions, and designed Figure 5. PM wrote the sections on RNA chaperones and tmRNA. SB and IU designed Figure 10. All authors approved the final version of the manuscript.

## Conflict of Interest

The authors declare that the research was conducted in the absence of any commercial or financial relationships that could be construed as a potential conflict of interest.

## Abbreviations

aa: amino acid
bp: base pair
MW: molecular weight
nt: nucleotide
RBS: ribosome binding site
sRNA: small RNA
SU: subunit
TA: toxin-antitoxin
TSS: transcription start site.
